# Roles of negative pressure wound therapy for scar revision

**DOI:** 10.3389/fphys.2023.1194051

**Published:** 2023-10-12

**Authors:** Xiaotong Qiu, Haoming Luo, Guobao Huang

**Affiliations:** ^1^ Affiliated Hospital of Weifang Medical University, School of Clinical Medicine, Weifang Medical University, Weifang, China; ^2^ Department of Burns and Plastic Surgery, Jinan Central Hospital, Jinan, China; ^3^ Department of Thyroid Head Neck and Maxillofacial Surgery, The Third Hospital of Mianyang & Sichuan Mental Health Center, Mianyang, China; ^4^ Department of Burns and Plastic Surgery, Central Hospital Affiliated to Shandong First Medical University, Jinan, China

**Keywords:** burns, negative pressure wound therapy, scar revision, skin transplantation, flap transplantation

## Abstract

The purpose of this study is to review the research progress of negative pressure wound therapy (NPWT) for scar revision and discuss the prospects of its further study and application. The domestic and foreign literatures on NPWT for scar revision were reviewed. The mechanism and application were summarized. NPWT improves microcirculation and lymphatic flow and stimulates the growth of granulation tissues in addition to draining secretions and necrotic tissue. As a significant clinical therapy in scar revision, NPWT reduces tension, fixes graft, and improves wound bed. In the field of scar revision, NPWT has been increasingly used as an innovative and constantly improving technology.

## 1 Introduction

Scar hyperplasia and contracture deformity bring serious harm to the patients’ physical and mental health. They damage appearances and affect functions of the patients, along with discomfort and unpleasant feelings such as itchiness, redness, pain, depression, and fear. Patients who have chronic ulcer and refractory wound in scar tissue are more likely to develop scar cancer.

Currently, scar revision methods include scar excision and skin grafting, composite skin grafting over human acellular dermal matrix scaffold, and expanded flaps. With the development of negative pressure wound therapy (NPWT), the effect of scar revision has been significantly improved. The use of NPWT in burn surgery has attracted much attention (Chai and Shen, 2015), and its concept of promoting wound repair has taken root in people’s hearts. However, little attention has been paid to its function in scar revision.

In recent years, NPWT has been recommended as a significant treatment for scar revision (Cai et al., 2017). The technique is simple and has a good prognosis. Since published studies on scar revision rarely present the effect of NPWT alone, this study also refers to wounds similar to scar revision.

## 2 NPWT materials

In NPWT, the materials used include a foam sponge, drainage tube, and semipermeable membrane. Two main types of foam are available; a polyurethane foam or a denser polyvinyl alcohol foam. Suction in negative pressure creates a clean and one-way sealed environment around the wound. The characteristics of foam sponges have been widely reported (Agarwal et al., 2019). The neglected semipermeable membrane is also related to effectiveness and comfort (Dooley et al., 2012). It is a one-way, breathable, transparent film, and its main components are polyurethane and acrylic. Oxygen can enter through the dressing, and water vapor and carbon dioxide leave the wound. Consequently, little infiltration occurs around the wound.

Several technical advances in NPWT devices and dressings have been made. The feasibility of home NPWT and single-use NPWT has been verified. (Mushin et al., 2017; Lim et al., 2021; Wilkinson et al., 2021). Other dressings may be considered for use in combination with NPWT. The gauze is placed over the wound to prevent the granulation from growing into the negative pressure material. Silver-ion dressings may increase the antibacterial effect of NPWT. In addition, the foam dressing is fixed around the negative pressure material to avoid tension blisters. Notably, some devices are only validated *in vitro*.

## 3 NPWT mechanism

### 3.1 Drain secretions and remove necrotic tissue

NPWT has been recognized for its remarkable effect on enhancing wound drainage and removing bacterial products (Agarwal et al., 2019). It is carried out in a closed environment to prevent cross-infection (Huang et al., 2021). In addition, the vascularized bed with a low degree of bacterial colonization promotes the likelihood that a skin graft would succeed (Kantak et al., 2016). The NPWT group had considerably higher CD34 and CD68 levels than the traditional group (Yang et al., 2021). It could play an important role in the inflammatory response of wound healing by effectively draining secretions and removing necrotic tissues.

### 3.2 Promote circulation and lymphatic return, reduce hematoma and edema

The pressure difference between the inside and outside of the capillaries and the endothelial intercellular space of lymphatic capillaries are both increased by the mechanical traction of negative pressure. In addition, it appears that the levels of blood supply and lymphatic return flow have increased. Furthermore, tissue edemas are reduced (Cagney et al., 2020).

Vascular endothelial growth factor and angiopoietin-2 (Ang-2) levels in the wound are much higher in NPWT, which may be caused by the closed and hypoxic environment (Ma et al., 2016; Yang et al., 2021; Zhu et al., 2021). Negative pressure provides effective and continuous power to the local circulation of the wound, reducing the seroma and hematoma, as compared with traditional dressing (Nagata et al., 2018; Mangelsdorff et al., 2019; Zwanenburg et al., 2020; Bueno-Lledo et al., 2021).

### 3.3 Stimulate granulation tissue growth and improve vascular bed conditions

Studies found that graft loss was associated with improper placement of skin grafts on an ill-prepared wound bed (Hsiao et al., 2017). NPWT improves wound healing by local immune modulation, hypoxia-mediated signaling and mechanoreceptors (Glass et al., 2014). Higher levels of cellular fibronectin (cFN) and transforming growth factor-β1 (TGF-β1) were expressed in the NPWT group compared to the traditional group, which stimulated granulation tissue formation (Yang et al., 2017).

Continuous negative pressure reduces the time needed to heal before skin grafting by drawing interstitial fluid from the wound, promoting the growth of capillaries and granulation tissue and improving blood circulation (Sun et al., 2021).

## 4 Clinical application of NPWT in scar revision

NPWT maintains negative pressure in the wound for wound treatment by attaching suction devices to specialized wound dressings. The use of NPWT in burn surgery has received a lot of interest. Its mechanism and efficacy in preventing infection, increasing blood supply, and reducing edema have all been demonstrated (Chai and Shen, 2015).

### 4.1 Reduce incision tension

Surgical site infections (SSIs) are usually accompanied by dehiscence of surgical wounds (Strickler et al., 2021). The negative pressure material and semipermeable membrane can transfer the incision tension to the surrounding skin. Less lateral force may resist mechanical stresses, delaying closure and predisposes wounds to dehiscence and infection. Smaller sponges, such as 3-cm-wide, should be considered to reduce incision tension and dehiscence (Googe et al., 2020).

Reconstruction of contracture scar often requires scar modification and flap transplantation. The tension of the incision increases after suturing the flap, which might cause skin marginal necrosis. The technique in NPWT reduces incision tension and improves blood flow in the flap, especially at the edge and tip of the wound. At the same time, the two sides of the incision were matched neatly to improve the healing quality. In 219 incision cases, Chai and Shen (2015) used the NPWT to reduce tension following scar resection, and there were no complications. Cai et al. (2017) combined the NPWT and scar excision to treat 25 burn children with hypertrophic scars. All incisions healed well without redness, effusion, and rupture.

According to a multinational, observer-blinded randomized controlled trial (RCT) involving 507 patients from 31 centers, the NPWT is effectively treats subcutaneous wound healing impairment following surgery (Seidel et al., 2020). The majority of wounds in the NPWT group were sutured. However, in the traditional group, there was a higher rate of wounds healed by secondary intention.

### 4.2 Prevent infection

SSIs are one of the most common postoperative complications (Bhangu et al., 2018). The use of NPWT in surgical incisions significantly resulted in a lower SSI risk at 30 days and 3 months postoperatively and reduced hospitalization costs (O'Leary et al., 2017; Javed et al., 2019; Hasselmann et al., 2020; Bueno-Lledo et al., 2021). The above wound is similar to the wound after scar resection, with high incision tension, which is characterized by easy rupture and infection. The effect of SSI prevention was more pronounced in the NPWT group when the incidence of SSIs was ≥20% in the traditional group (Meyer et al., 2021).

Notably, not all surgical incisions benefit from NPWT (Gabriel et al., 2019). Tuuli et al. (2020) observed no significant difference in SSI risk reduction between the NPWT group and the conventional dressing group in a RCT of 1,608 obese women undergoing cesarean delivery. A meta-analysis involving 792 patients from five RCTs also reported conflicting results. The study concluded that the current evidence does not support the efficacy of routine NPWT to prevent SSIs (Kuper et al., 2020).

Many factors, such as the procedure type, wound classification, negative pressure device, and parameters, contribute to the above differences. NPWT for different wounds should be cautiously adopted, and the available incision management plans must assess and address each case. Furthermore, several RCTs are in progress, and we await their results.

### 4.3 Fix graft and promote survival

Skin grafts are widely used to repair large skin defect following scar release and resection. However, there are problems with the traditional way to dress the wound, that is, there are instances of uneven pressure, improper tension, and insufficient drainage, especially on uneven or mobile surfaces, such as neck and joint. NPWT is an effective way to fix skin grafts following scar resection, resulting in proper pressure, stable tension, and adequate drainage of the graft area. It has also demonstrated significant advantages in reducing wound infection, healing time, and hospital stay (Li et al., 2017).

NPWT produces promising results (Nakamura et al., 2018; Nakamura et al., 2021; Pedrazzi et al., 2021). Improvement of blood circulation promotes the survival of the skin graft. Furthermore, NPWT can treat potentially infected wounds and reduce the duration of antibiotic therapy. Thus, NPWT significantly increased graft survival, and reduced the incidence of reoperation because of skin graft failure (Yin et al., 2018; Sun et al., 2021). It is successfully applied to keep the graft immobilized, especially in exudative, irregular, and muscle-exposed wounds and special anatomical sites, with no serious wound-related adverse effects observed. In some cases, it is highly recommended that the gauze isolate the graft from a foam sponge, such as muscle-exposed wounds (Nakamura et al., 2021). It prevents difficulty in detaching when the NPWT sponge was removed.

NPWT reduces surgery time by saving fixation after skin transplantation. Furthermore, NPWT can be applied to secure grafts without any sutures or staples (Inatomi et al., 2019). It means that pain, staple retention, and complications associated with this procedure were avoided.

Simao (2020) developed a simple dressing for applying negative pressure after skin grafting. It is mainly made with three layers: petrolatum gauze soaked in ointment, gauze pad, and waterproof transparent film. All the air is aspirated using a 20 cc syringe, after fixing the dressing on the graft. In this way, effective fixation and pressure can last up to 5–7 days. However, the lack of drainage, display, and adjustability of negative pressure parameters is a downside.

The U-shaped form fashioned by researchers was applied over the suture line. Its opening was at the root of the vessel after flap reconstruction. Consequently, the vascular pedicle may be kept from compressing. And the condition and temperature of the flap could be monitored (Chen et al., 2021). This innovative modification of eliminates the concern that NPWT affects blood flow in the vascular pedicle.

### 4.4 Preparation before skin transplantation to improve the success rate of surgery

The wound surface following scar release or resection is often uneven, which needs to be covered by flaps or skin grafts. NPWT before transplantation can improve the survival rate, especially when the condition of the wound bed is not ideal. At the same time, the compression of the negative pressure dressing may also tighten the wound edge and reduce the extent of the wound, ultimately reducing the area of skin grafting (Huang et al., 2021).

Scar reconstructive wounds have few evidence on NPWT repairing wound beds; however, other wounds of similar types have been reported. In the treatment of necrotizing fasciitis and chronic venous leg ulcers (CVLUs), NPWT can be used as a wound bed preparation (Ren et al., 2020; Zhang B. R. et al., 2021). Common complications were effectively reduced on the account of applying NPWT before and after skin grafting in electrical burns and diabetic foot wounds (Smuđ-Orehovec et al., 2018; Gomez-Ortega et al., 2021).

Patients using NPWT might experience fewer SSI during primary closure of surgical wounds (Norman et al., 2020). By using an emergency delay method and NPWT, Ishii et al. (2020) were able to successfully salvage a severely congested propeller perforator flap. Interestingly, the flap had transferred to the donor site for some time. Then, it was retransferred to the defect on day 19 after the wound bed was prepared using NPWT. After flap necrosis in the primary operation, Gigliotti et al. (2021) prepared the wound bed for the second operation combined with debridement, antibiotics, and NPWT. And there was 100% viability for above retransplanted flaps.

### 4.5 Reduce dressing and pain

The wound is maintained relatively clean and moist because of NPWT. Replacement of the dressings was reduced, which decreases pain experience and the workload of medical staff. The economic burden and the length of hospital stay were also cut down (Hsiao et al., 2017; Yin et al., 2018). Especially, children have a low pathophysiological pain tolerance. NPWT reduces pain, which helps children comply more readily (Huang et al., 2021). The NPWT is a reliable, simple procedure with an excellent clinical utility and feasibility.

NPWT significantly reduces the donor site pain (Kantak et al., 2017). It may be related to the good fixation of the negative pressure dressing and the reduced shear force of the traditional dressing. In the meantime, NPWT promotes reepithelialization, accelerates healing, and reduces scar formation. In addition, the moist wound environment is the first choice for healing at the donor site. NPWT was found to lower the occurrence of flap donor sites significantly (Mangelsdorff et al., 2019).

### 4.6 Reduce secondary scar

NPWT can promote wound healing after scar resection and reduce secondary scars. This advantage distinguishes it from traditional dressings. Preclinical studies have shown that NPWT increases wound strength and reduces scar width (Zwanenburg et al., 2021). After scar removal and NPWT application, the appearance, function, and comfort of the children all clearly improved (Cai et al., 2017). Furthermore, the scar area was significantly reduced, ranging from 36% to 100% 6 months after the surgery. NPWT uses a simple and effective device that improves the appearance and histochemical properties of incision scars. Its effective fixation and compression can reduce collagen deposition and scar formation (Nagata et al., 2018).

NPWT improved the smoothness of the scar formed after skin grafting and the satisfaction of the patients and researchers with the scar (Mo et al., 2021; Zwanenburg et al., 2021). Unlike conventional fixation techniques, NPWT applies negative pressure between the graft and the recipient, removing space and attracts the entire graft with uniform pressure. The possible reason is that NPWT provided a more uniform pressure and prevention of shear force, resulting in a uniform thickness of scar tissue. The surface of the scar is more regular and flatter.

### 4.7 Complications

NPWT may be more likely to develop skin blisters than standard dressing (Kuo et al., 2021; Norman et al., 2022). It recovers on its own in approximately 1 week. Appropriately changing the shape of the NPWT dressing can reduce the formation of tension blisters on the edge of the dressing (Zhang C. et al., 2021), because it can avoid the gap between the dressing and the skin when a semipermeable membrane is attached.

Importantly, inappropriate use of NPWT might result in severe complications such as skin necrosis, bleeding, and allergic reactions (Agarwal et al., 2019; Ji et al., 2021). Medical staff should observe the effect of NPWT. Once these phenomena occur, NPWT must be stopped.

## 5 Parameter of NPWT in scar revision

The optimum negative pressure of NPWT create a favorable environment for wound healing (Horch et al., 2020). It is generally believed that 125 mmHg provides the most conducive environment for granulation tissue growth and blood supply. Zhu et al. (2021) also showed that an environment with a pressure of 125 mmHg in NPWT could accelerate bone regeneration. A single pressure setting throughout may not be the best choice for all wounds.

In recent years, the setting of low negative pressure has attracted much attention. A systematic review suggested that high negative pressure may cause the ineffectiveness of NPWT on graft survival (Shimada et al., 2022). A lower negative pressure, such as 75 mmHg, is ideal for initial engraftment because it promotes strong adherence between the skin graft and the wound bed (Maruccia et al., 2017).

Other factors that are easily ignored when setting negative pressure parameter include age and constitution. Adult devices and NPWT parameters have been adapted to pediatric use (de Jesus et al., 2018). Extra care is needed to protect the delicate tissues of pediatric or weak patients. The negative pressure should be reduced appropriately, and it is not >75 mmHg.

Compared with the continuous mode, the intermittent mode significantly promotes wound healing. But it also increases pain experience. The circulatory mode activates, changing the circulatory within a certain range of negative pressure. The curative effect is comparable to intermittent mode, but the pain is significantly reduced.

It should be noted that NPWT dressings are challenging to apply to areas without sufficient healthy skin (Jiang et al., 2021). For irregular wounds, it is difficult to maintain appropriate negative pressure. In addition, NPWT may leak air due to patients’ movement and perspiration, affecting the treatment effect.

The new monitoring equipment mainly focuses on *in vitro* studies, demonstrating its application potential. A noninvasive system was designed for adjusting the NPWT parameters (Wilkinson et al., 2021). Bioreactors, which evaluate the effect of NPWT on skin anatomy and physiology, also helpin parameter adjustment. (Notorgiacomo et al., 2022).

## 6 Summary and prospect

There are few articles summarizing the research of NPWT for scar revision. Although there are few separate literatures, NPWT is sometimes used as an important supplementary method in the traditional research of scar revision. The role of NPWT in other similar wounds may also be beneficial for patients with scar revision.

There was no significant increase in wound-related adverse events with NPWT compared with conventional care. Complications can be prevented by appropriate measures. In recent years, the cost of NPWT has been reduced, which relieves economic burden of patients. And it is worthy of clinical promotion. In addition, more studies are needed to elucidate the mechanism of NPWT in scar revision.

Future research should examine fixation time and observation time to find a better option for parameter, so as to provide the basis for the guidelines.
